# Dominant viral pathologies in the extensive and semi-intensive animal breeding and their treatment mode in ethno veterinary medicine in Benin

**DOI:** 10.14202/vetworld.2015.1424-1434

**Published:** 2015-12-23

**Authors:** T. M. Kpodékon, C. A. Ogni, H. Dassou, T. J. Dougnon, C. Boko, G. B. Koutinhouin, J. S. E. Goussanou, A. Akoegninou, I. Youssao

**Affiliations:** 1 University of Abomey-Calavi (UAC), Polytechnic School of Abomey-Calavi (EPAC), Department of Animals Health and Production, Research Laboratory of Applied Biology (LARBA), 01 BP 2009 Cotonou, Benin; 2 University of Abomey-Calavi (UAC), Faculty of Sciences and Technology, Department of Plant Biology, Laboratory of Botany and Plant Ecology, 01 BP 4521 Cotonou, Benin

**Keywords:** Benin, ethnoveterinary survey, medicinal plants, viral diseases

## Abstract

**Aim:**

This study aims to identify the dominant viral animal pathologies and to list the traditional recipes used by the breeders for their treatment.

**Materials and Methods:**

The method of data collection was based on a retrospective survey. Thus, 787 breeders and agro-breeders scattered in the eight agro-ecological areas of Benin were interviewed using semi-structured questionnaires.

**Results:**

In total, 5 pathologies were reported by breeders. Among those pathologies, foot and mouth disease was reported by all of the breeders of the southern part of Borgou compared with the other areas (p<0.05) and treated by 25 species of medicinal plants. African swine fever was the main pathology reported (22.92%) (p<0.05) in the fishery areas which is controlled by 7 medicinal plants. Pseudorinderpest was more reported (33.78%) (p<0.05) in the cotton area of central Benin and treated by 8 medicinal plants. There is also Newcastle disease that was mostly reported in the Western Atacora and treated by 32 medicinal plants as well as fowl pox which was a more reported in the lands of the bar area and the low-pressure area about 34.48% and 36.17% proportions, respectively, and treated by eight medicinal plants.

**Conclusion:**

The breeders in Benin possess rich ethno veterinary knowledge on medicinal plants and their uses in the treatment of livestock. A total of 57 medicinal plants have been inventoried to fight against five major viral diseases as African swine fever, pseudorinderpest and foot and mouth disease. The common plants used to treat viral disease in general were *Euphorbia unispina*, *Euphorbia poissonii*, *Lannea acida*, and *Mangifera indica*. The most harvested organs on the plants reported in this survey were the barks, the leaves, and the whole plants. To better develop our indigenous resources, it would be important to expand this ethno-pharmacological investigation to other diseases category.

## Introduction

Animal rearing is one of the main activities carried out by humans (33%) after crops production (42%) to satisfy their needs and ensure food security [1]. However, there are certain limiting factors restricting the development of livestock. It has been established that animal diseases are a major constraint to livestock production.

Viral infections are one of the most transmissible diseases in the world. Certain breeders resort to available antiviral agents which have shown the good result on viral diseases. However, resource-poor breeders in rural and peri-urban areas have limited access to veterinary care in terms of support services, information about the prevention and treatment of livestock diseases, and preventative and therapeutic veterinary medicines [2]. According to the FAO [3], the lack of drugs to treat diseases and infections causes losses of 30-35% in the breeding sector of many developing countries, where poor animal health remains the major constraint to breeding. These are significant reasons for breeders to appropriate ethnoveterinary medicine. Hence, many breeders in developing countries still rely on medicinal plants and traditional healing practices for daily healthcare needs of their animals, in spite of the advancement in conventional medicine [4].

In many parts of the world, ethnoveterinary medicine is frequently used to treat and to control animal diseases in livestock by the breeders because it is easily accessible compared to conventional drugs, easy to prepare and administer, and cost very little or nothing at all [5-7]. Ethnoveterinary medicine is defined as a traditional knowledge, folk beliefs, skills, methods, and practices used for the treatment of livestock ailments [8]. The role of ethnoveterinary in livestock development is beyond dispute. During the 32^nd^ session of UNESCO, ethnoveterinary has been recognized as one of the important components of indigenous cultural heritage that need to be studied, sustained and protected [9]. Thus, the expenses for the use of traditional and complementary alternative medicine are exponentially growing in many parts of the world. In the countries as Kenya, Italy, Morocco, Spain, Egypt, Greece, Algeria, Canada, South Africa, Pakistan, Uganda, Brazil, Argentina, India, Nigeria and Germany, scientific studies and documentation of indigenous knowledge on ethnoveterinary medicinal plants have been initiated. In addition, considering the cases of resistance to the conventional drug used around the world [10], it is necessary to resort the medicinal plants use by breeders in livestock. Most ethnoveterinary studies focus almost exclusively on all group of pathologies, but few research were focused only on ailments specific group. Like most African countries, Benin is an important repository of biological diversity, but there are limited documentation and research on animal production, diseases treatment, and ethnoveterinary knowledge. In view to value medicinal plant knowledge, the ethno-pharmacological investigation focused on parasitic diseases [11] and bacterial diseases [12] were conducted in Benin.

The aim of this study was to identify the most important viral diseases of animal livestock in the eight agroecological zones of Benin. It also aims to identify and document medicinal plants used by the breeders to treat and control these diseases.

## Materials and Methods

### Ethical approval

The study protocol was approved by the Department of Health and Animal Production (DHAP) and National Herbarium at Abomey-Calavi University. The diseases identification has been done in collaboration with veterinary inspector. The plants samples were collected and identified by botanist experts.

### Study area

**Figure-1:**
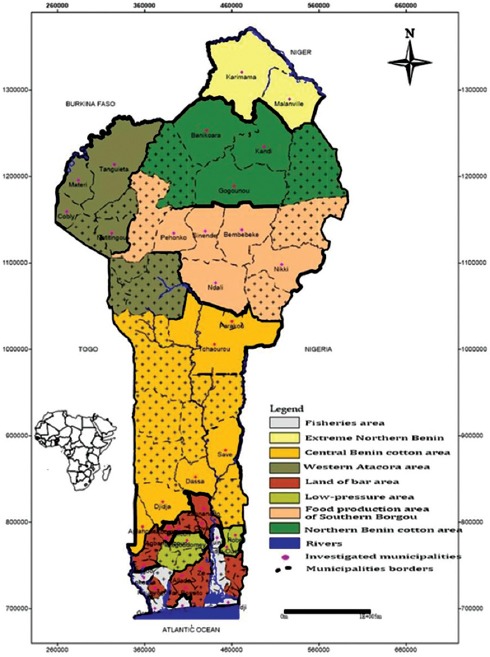
Study area.

The present study was undertaken in a total of eight agroecological zones of Benin (Figure-1). In each agro-ecological area, municipalities were selected using the statistic of livestock registered according to Country Stat. A total of 38 municipalities were chosen as it is shown in the Figure-1. Each livestock included in the study was selected according to breeders that reared above 20 animals (all species).

### Data collection

To collect data, a retrospective study was undertaken from June to December 2013. The technic of memory recall was used to collect data on the past events that breeders experienced. Information on respondent characteristics, pathologies occurring in livestock from 2009 to 2013, origin of the main viral pathologies, the period in which the infection occurred, traditional treatment used by the breeders to prevent or treat diseases of livestock was obtained through semi-structured questionnaires. Animal pathologies occurring in the livestock were registered based on the description reported by the breeders. For each plant identified by the breeder, data were collected on organ used, methods of preparation, dosages, and routes of administration. In the laboratory, identification of the plants was done using the relevant taxonomic literature especially the analytics flora of Benin of Akoegninou *et al*. [13], Voucher specimens were deposited at the National Herbarium of the University of Abomey-Calavi in Benin.

### Statistical analysis

Collected data were encoded and stored in a database designed in Excel table. The Statistical analysis was performed using SAS [14]. To determine main diseases, the citation frequency methodology was used. The number of citation of specific disease was determined according to the number of time this disease was reported by breeders during the survey. The formula used to calculate the frequency citation was:


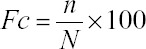


Where Fc is citation frequency; n is citation number of specific disease and N is total number of diseases citation.

To assess distribution of viral diseases, frequency citation was used, and variance analysis was performed taking into account agro-ecological areas. The F test was performed to measure any significant difference between frequencies of citation of a specific viral disease according to agro-ecological areas. The mean values were calculated and compared by agro-ecological zone using the Student’s *t*-test.

To calculate the frequency of each medicinal plant or traditional recipes used by breeders for the diseases treatment according to agroecological area, *Proc freq* procedure of STATA was used. The Chi-square (χ²) test was used to assess agroecological areas effect. The frequencies were compared to each other by using the bilateral Z test. For each relative frequency, confidence interval (CI) at 95% was calculated using the formula:


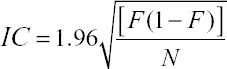


F is the relative frequency and N the sample size.

The histogram and diagram of the most plants and organ used were performed using the computer program Excel.

## Results

### Distribution of the viral pathologies

Though several viral diseases types were recorded as an animal health problem, a total of five viral diseases were identified by the breeders investigated. Diseases identified by the breeders were presented in Table-1. The most common viral diseases according to breeders in Extreme Northern Benin were foot and mouth disease at 97.06%. The Cotton area of Northern Benin and Cotton area of Central Benin were the most common characterized, respectively, by foot and mouth disease at 71.70% and 37.84%, followed by pseudorinderpest at 13.21% and 33.78%, and by Newcastle disease at 11.32% and 13.51%. An investigation conducted in the Food producing area of Southern Borgou showed that foot and mouth disease (100%) were the livestock diseases frequently observed by the breeders. Newcastle disease (48.10%), foot and mouth disease (27.85%), and African swine fever (22.78%) were frequently registered in the West-Atacora area. Likewise, livestock diseases mainly registered in the Lands of Bar area were fowl pox (34.48%), pseudorinderpest (24.14%), Newcastle disease, and foot and mouth disease at 18.97% for each. In the low-pressure area, diseases frequently reported were fowl pox (36.17%), Newcastle disease (27.66%), and foot and mouth disease (21.28%). Concerning fisheries area, Newcastle disease, African swine fever, and fowl pox were the most common disease.

**Table-1:** Distribution of the dominant viral animal pathologies in the agro-ecological areas of Benin.

Pathologies	Animal species	Zone 1 (n=34)	Zone 2 (n=53)	Zone 3 (n=32)	Zone 4 (n=79)	Zone 5 (n=74)	Zone 6 (n=58)	Zone 7 (n=47)	Zone 8 (n=48)	Test of significance
Foot and mouth disease (%)	Cattle	97.06^b^	71.70^c^	100.00^a^	27.85^e^	37.84^d^	18.97^f^	21.28^f^	16.67^f^	***
Newcastle disease (%)	Poultry	0.00^e^	11.32^d^	0.00^e^	48.10^a^	13.51^cd^	18.97^c^	27.66^b^	25.00^b^	***
African swine fever (%)	Pig	0.00^d^	0.00^d^	0.00^d^	22.78^a^	1.35^c^	3.45^b^	2.13^bc^	22.92^a^	*
Pseudorinderpest (%)	Cattle, goat, sheep	2.94^d^	13.21^c^	0.00^f^	1.27^e^	33.78^a^	24.14^b^	12.77^c^	14.58^c^	***
Fowl pox (%)	Poultry	0.00^e^	3.77^d^	0.00^e^	0.00^e^	13.51^c^	34.48^a^	36.17^a^	20.83^b^	***

The percentages of the same line followed by different letters, differ significantly at the threshold of 5%.

*** p<0.001;

* p<0.05, Zone 1=Extreme north Benin; Zone 2=Cotton area of north Benin: Zone 3=Food producing area of south Borgou; Zone 4=West-Atacora area; Zone 5=Cotton area of central Benin; Zone 6=Lands of bar area; Zone 7=Low-pressure area; Zone 8=Fisheries area

About distribution of disease between agroecological areas, this study revealed that foot and mouth disease was more reported by all of the breeders in food producing area of Southern Borgou. The highest value of Newcastle disease was registered in the West Atacora area (48.10%) and the lowest value in Extreme Northern Benin. African swine fever was most reported in West-Atacora area (22.78%) and in fisheries area (22.92%). This disease was less observed in Extreme Northern Benin, Cotton area of northern Benin, and Food producing area of Southern Borgou. Concerning pseudorinderpest, the highest values were reported in the Cotton area of Central Benin and in lands of bar area, respectively, at 33.78% and 24.14%. Fowl pox related to poultry was more frequently revealed in the low-pressure area (36.17%) followed by lands of bar area (34.48%).

### Treatment of viral diseases

**Figure-2:**
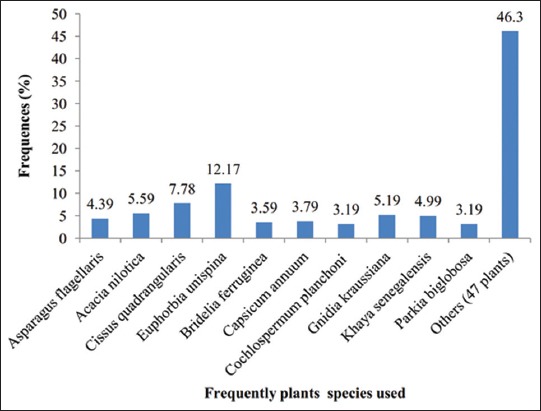
Frequently plants used to control viral disease.

During the field survey, 57 plant species of ethno-pharmacological importance were gathered throughout the study area (Tables-2-6) as having medicinal properties against five ailments. 22 plants were used to control foot and mouth disease; 35 for Newcastle disease; 7 for African swine fever and 8 respectively for pseudorinderpest and fowl pox. These medicinal plants were distributed among 34 botanical families. Most of the plants used belong to the family of Leguminosae-Mimosoideae (10.53%), *Euphorbiaceae* (8.77%). However, *Anacardiaceae*, *Asteraceae*, *Combretaceae*, *Lamiaceae*, Leguminosae-Papilionoideae, and *Solanaceae* were represented each at 5.26%. Among the main plants (Figure-2) used to control animal’s viral ailments, *Euphorbia unispina* was most reported by the breeders. Others plants, such as *Euphorbia poissonii*, *Lannea acida*, and *Mangifera indica*, were also mentioned during the survey.

**Table-2:** Remedies used for the treatment of foot and mouth disease.

Disease	Local name of plant	Scientific name of plant (used part)	Other combination	Preparation method	Dose	Route of administration	Posology	Time of treatment	Frequency
Foot and mouth disease	Boodouhi (P)	*Erythrina senegalensis* DC. (AA 6591)+*Bombax costatum* Pellegr. and Vuillet. (AA 6567) (root)	Ox urine	Maceration	-	Rinsing	Once a day	At will	3
	Banounoui (P)	*Pterocarpus erinaceus* Poir. (AA 6586) (bark)	Potash	Decoction	-	Rinsing	Twice a day	At will	1
	Hissi-hissi (F)	*Ocimum americanum* L. [AA 6582] (leaves)	-	Trituration	-	Rinsing	Twice a day	At will	1
	Toufalderehi (P)	*Entada africana* GuilI. and Perr. [AA 6582] (bark)	Potash	Decoction	-	Rinsing	Once a day	At will	2
	Gbagbanati (P)	*Boerhavia diffusa* (Kunth) Baker [AA 6599] (bark)	-	Decoction	-	Oral	-	-	2
	-	*Acacia nilotica* (L.) Willd. ex Delile ssp. nilotica (AA 6587) (seeds)	Urine	Maceration	-	Rinsing	Twice a day	1 month	4
	Naréhi (P)	*Parkia biglobosa* (Jacq.) R.Br. ex Benth. [AA 6590](fruit)	-	Trituration	-	Oral	Once a day	1 week	4
	Nanwankou	*Sorghum bicolor* (L.) Moench (AA 6601) (grain)	-	Infusion	-	Oral	Once a day	1 week	5
	Gaoure or Tchabi (P)+ Kahi (P)+Ganeï (P)	*Lannea acida* A. Rich. s.l. (AA 6554)+*Khaya senegalensis* (Desr.) A. Juss. [AA 6596]+*Detarium mespiliformis* Hochst. Ex A.DC. (AA 6574) (bark)	-	Infusion	-	Oral and Rinsing	Once a day	1 week	3
	-	*Vitellaria paradoxa* C.F.Gaertn. ssp. paradoxa [AA 6604] (bark)+*Pterocarpus erinaceus* Poir. [AA 6586] (bark)+*Acacia nilotica* (L.) Willd. ex Delile ssp. nilotica (AA 6587) (root)	-	Decoction	1 L adult and ½ L for young	Oral	Once a day	3 days	1
	-	*Anogeissus leiocarpa* (DC.) Guil. and Perr. (AA 6570) (bark)	-	Powder	At will	Oral	Once on 2 days	1 week	1
Foot and mouth disease	-	*Detarium microcarpum* Guill. and Perr. [AA 6584] (leaves)	Salt	Trituration	-	Oral	Once a day	3 days	2
	-	*Balanites aegyptiaca* (L.) Delile [AA 6564] (bark)	Salt	Powder	-	Oral	Twice a day	At will	5
	-	*Kigelia africana* (Lam.) Benth. [AA 6565] (Fruit)	-	Decoction	½ L per animal	Oral	Once a day	3 days	2
	-	*Cassia sieberiana* DC. [AA 6583] (bark)	-	Decoction	-	Oral	Once a day	3 days	3
	Sôtô-Djabi (P)	*Agelanthus dodoneifolius* (DC.) Polh. and Wiens [AA 6594] (leaves)	-	Fumigation	-	External	Once a day	2 days	3
Foot and mouth disease	Sorokouhi (P)	*Pericopsis laxiflora* (Benth. ex Baker) Meeuwen (AA 6592) (bark)	Salt	Powder	-	Oral	Once/week	At will	6
	Loogoh (P)	*Manihot esculenta* Crantz (AA 6578) (leaves)	Fèces de caprins	Fumigation	-	External	Once a day	1 month	2
	Tchami (P)	*Lannea acida* A.Rich. s.l. [AA 6554] (root)	Hen egg+salt	Calcination de la root	-	Oral	Once a day	2 weeks	2
	Bênahi+Sôtô (P)	*Saba comorensis* (Boj.) Pichon [AA 6558] (bark)+*Agelanthus dodoneifolius* (DC.) Polh. and Wiens (AA 6594) (leaves)	Hen egg	Trituration	-	Oral	Twice a day	At will	2
	Kahi (P)	*Khaya senegalensis* (Desr.) A. Juss. (AA 6596) (bark)	Ox urine	Maceration	-	External/Oral	Twice a day	1 week	10
	Gade (P)	*Acacia nilotica* (L.) Willd. ex Delile ssp. nilotica [AA 6587] (fruit)	-	Maceration	-	Rinsing	Twice a day	At will	23
	Tiguerehi (P)	*Terminalia mollis* M. A. Lawson (AA 6572) (bark and root)	-	Decoction	-	Rinsing	Twice a day	3 days	4

**Table-3:** Remedies used for the treatment of Newcastle disease.

Disease	Local name of plant	Scientific name of plant (used part)	Other combination	Preparation method	Dose	Route of administration	Posology	Time of treatment	Frequency
Newcastle disease		*Euphorbia unispina* N.E.Br. [AA 6577] (Whole plant)	-	Maceration	-	Oral	Once a day	1 week	3
	-	*Entada wahlbergii* Harv. [AA 6589] (bark)	-	Maceration	-	Oral	Twice a day	At will	1
	-	*Gnidia kraussiana* Meisn. [AA 6608]+*Euphorbia unispina* N.E.Br. [AA 6577] (whole plant)	-	Maceration	-	Oral	One to four times	At will	4
	-	*Capsicum annuum* L. [AA 6605] (fruit)	-	Trituration	-	Oral	Once a day	1 day	2
	-	*Asparagus flagellaris* (Kunth) Baker [AA 6593] (leaves)	-	Maceration	-	Oral	One to four times	At will	5
	-	*Capsicum annuum* L. [AA 6605] (bark)	-	Maceration	-	Oral	-	-	7
	Ira (I)	*Bridelia ferruginea* Benth. [AA 6575] (bark)	-	Maceration	-	Oral	-	-	13
	Pampale (P)	*Sapium grahami* (Stapt) Prain [AA 6579] (bark)	-	Maceration	-	Oral	-	-	2
Newcastle disease	Sesera (P)	*Euphorbia poissonii* Pax [AA 6576] (stem)	Red pepper	Maceration	-	Oral	-	At will	10
	Tambo (I)	*Piper guineense* Schumach. and Thonn. [AA 6600] (fruit)	Potash	Decoction	1 L (adult) and ½ L (young)	Oral	Once a day	3 days	1
	Mango (F)	*Mangifera indica* L. [AA 6555]+*Anacardium occidentale* L. [AA 6553] (bark)	-	Maceration	-	Oral	-	-	2
		-	*Cissus quadrangularis* L. [AA 6609] (whole plant)	-	Maceration	-	Oral	Once a day	1 week	9
		-	*Khaya senegalensis* (Desr.) A.Juss. [AA 6596] (bark)	-	Maceration	-	Oral	Once a day	1 week	10
		-	*Euphorbia unispina* N.E.Br. [AA 6577](whole plant)	-	Maceration	-	Oral	3 to 4 times/day	At will	28
		Nare (P)	*Parkia biglobosa* (Jacq.) R.Br. ex Benth. [AA 6590] (bark)	-	Maceration	-	Oral	Once a day	1 week	6
		-	*Nicotiana tabacum* L. [AA 6607] (whole plant)	-	Maceration	-	Oral	Once a day	1 week	2
Newcastle disease	-	*Bombax costatum* Pellegr. and Vuillet [AA 6567] (serve)+*Adenium obesum* (Forsk.) Roem. and Schult. [AA 6557] (stem)+*Isoberlinia doka* Craib and Stapf [AA 6585] (bark)	-	Decoction	-	Oral	Once a day	At will	1
	-	*Vitellaria paradoxa* C.F.Gaertn. sp. paradoxa [AA 6604]+*Parkia biglobosa* (Jacq.) R.Br. ex Benth. [AA 6590] (bark)	-	Maceration	-	Oral	Once a day	1 week	4
	-	*Anogeissus leiocarpa* (DC.) Guil. and Perr. [AA 6570] (bark)+*Euphorbia unispina* N.E.Br. [AA 6577] (whole plant)	-	Maceration	-	Oral	Twice a day	1 week	2
	-	*Lannea acida* A.Rich. s.l. [AA 6554] (bark)+ *Euphorbia unispina* N.E.Br. [AA 6577] (whole plant)	-	Maceration	-	Oral	Once a day	1 week	9
	-	*Pteleopsis suberosa* Engl. and Diels [AA 6571] (bark)	-	Maceration	-	Oral	3 to 4 times/day	At will	5
	-	*Gnidia kraussiana* Meisn. [AA 6608] (bark)	-	Maceration	-	Oral	Once a day	At will	8
	-	*Asparagus flagellaris* (Kunth) Baker [AA 6593] (tuber)	-	Maceration	-	Oral	Once a day	At will	10
	-	*Gnidia kraussiana* Meisn. [AA 6608]+*Euphorbia unispina* N.E.Br. [AA 6577] (whole plant)	-	Maceration	-	Oral	3 to 4 times	At will	8
	-	*Mitracarpus hirtus* (L.) DC. [AA 6602]+ *Euphorbia unispina* N.E.Br. [AA 6577] (whole plant)	-	Maceration	-	Oral	Once a day	1 week	2
Newcastle disease	Ehouzou (F)	*Chromolaena odorata* (L.) R.M.King [AA 6562] (leaves)	-	Trituration	At will	Oral	-	At will	5
	-	*Ocimum basilicum* L. [AA 6580] (leaves)+ *Capsicum annuum* L. [AA 6605] (fruit)+ *Garcinia kola* Heckel [AA 6568] (fruit)	-	Trituration	½ bamboo glass	Oral	Once a day	2 days	5
	Kpatin yovo (F)	*Moringa oleifera* Lam. [AA 6598] (fruit)	-	Maceration	1 drop	Oral	Once a day	2 days	3
	Mangoti (F)	*Mangifera indica* L. [AA 6555] (bark)	-	Maceration	At will	Oral	-	At will	7
	Gninsikin (F)	*Momordica charantia* L. [AA 6573] (whole plant)	-	Maceration	-	Oral	Twice a day	At will	5
	-	*Annona senegalensis* Pers. ssp. Senegalensis [AA 6556] (root)+ *Capsicum annuum* L. [AA 6605] (fruit)	-	Maceration	At will	Oral	-	At will	3
	-	*Khaya senegalensis* (Desr.) A.Juss. [AA 6596]+ *Cassia sieberiana* DC. [AA 6583] (bark)	-	Maceration	½ to 1 L	Oral	-	At will	2
Newcastle disease	Aloman (F)	*Vernonia amygdalina* Delile [AA 6563]+ *Ocimum gratissimum* L. [AA 6581] (leaves)	Ampicilline	Trituration	At will	Oral	Twice a day	At will	6
	-	*Cochlospermum planchonii* Hook.f. [AA 6569] (root)	-	Maceration	-	Oral	Once a day	At will	3
	-	*Pericopsis laxiflora* (Benth. ex Baker) Meeuwen [AA 6592] (bark)+*Cissus quadrangularis* L. [AA 6609] (whole plant)	-	Maceration	-	Oral	Once a day	1 month	9
	Koyanre+ booga (P)	*Cissus quadrangularis* L. [AA 6609] (whole plant)+*Sorghum bicolor* (L.) Moench [AA 6601] (stubble)	-	Maceration	-	Oral	Once a day	At will	6
	Nare+ sisiridji (P)	*Parkia biglobosa* (Jacq.) R.Br. ex Benth. [AA 6590]+*Anacardium occidentale* L. [AA 6553] (bark)	-	Maceration	-	Oral	Once a day	1 week	2

**Table-4:** Remedies used for the treatment of African swine fever.

Disease	Local name of plant	Scientific name of plant (used part)	Other combination	Preparation method	Dose	Route of administration	Posology	During of treatment	Frequency
African swine fever	-	*Cissus quadrangularis* L. [AA 6609] (bark)	-	Maceration	-	Oral	Once a day	At will	4
	-	*Adansonia digitata* L. [AA 6566] (fruit)	-	Decoction	-	Oral	Once a day	At will	1
	Tchrotchi (F) (Gninsikin)	*Momordica charantia* L. [AA 6573] (whole plant)	-	Fourrage	At will	Oral	3 times/day	At will	1
	-	*Cochlospermum planchonii* Hook.f. [AA 6569] (root)	-	Maceration	-	Oral	Once a day	1 week	13
	-	*Datura metel* L. [AA 6606] (leaves)	Potash	Decoction	-	Oral	Once a day	1 week	3
	-	*Cussonia arborea* Hoehst. ex A. Rich. [AA 6560] (seeds)	-	Pilage	Pinch	Oral	Twice a day	At will	1
	-	*Ageratum conyzoides* L. [AA 6561] (whole plant)	-	Fourrage	-	Oral	3 to 4 times/day	At will	6

**Table-5:** Remedies used for the treatment of the pseudorinderpest.

Disease	Local name of plant	Scientific name of plant (used part)	Other combination	Preparation method	Dose	Route of administration	Posology	During of treatment	Frequency
Pseudo-rinderpest	Baadoman (P)	*Momordica charantia* L. [AA 6573] (leaves)	-	Trituration	-	Oral	Twice a day	At will	2
	Ira (I)	*Bridelia ferruginea* Benth. [AA 6575] (bark)	Potash	Maceration	-	Oral	-	-	5
	Ganahi (P)	*Ficus sur* Forssk. [AA 6597] (fruit)	-	Maceration	½ L	Oral	Once a day	-	2
		*Asparagus flagellaris* (Kunth) Baker [AA 6593] (tuber and leaves)	-	Maceration	-	Oral	Once a day	1 week	7
		*Cissus quadrangularis* L. [AA 6609] (leaves)	-	Maceration	-	Oral	Once a day	1 week	5
	Botouri+ barkou (P)	*Gnidia kraussiana* Meisn. [AA 6608] (leaves)+ *Cissus quadrangularis* L. [AA 6609] (whole plant)	-	Maceration	-	Oral	3 to 4 times/day	At will	6
		*Sida garckeana* Pol. [AA 6595] (leaves)	-	Trituration	-	Badigeonnage	Twice aday	At will	3
		*Euphorbia unispina* N.E.Br. [AA 6577] (whole plant)	-	Maceration	-	Oral	Once a day	1 day	5

**Table-6:** Remedies used for the treatment of the fowl pox in Benin.

Desease	Local name of plant	Scientific name of plant (used part)	Other combination	Preparation method	Dose	Route of administration	Posology	During of treatment	Frequency
Fowl pox	Aloman (F)	*Vernonia amygdalina* Delile [AA 6563] (leaves)	-	Trituration	-	Oral	Twice a day	3 days	3
	Nyinsikin (F)	*Momordica charantia* L. [AA 6573] (whole plant)	-	Trituration	-	Oral	Twice a day	At will	6
	kessou-kessou (F)	*Ocimum americanum* L. [AA 6582] (leaves)	-	Trituration	-	Badigeonnage	Twice a day	-	4
	Yébésé (M)	*Capsicum annuum* L. [AA 6605] (fruit)	-	Pilage	-	Oral	-	2-3 weeks	2
	Lobokle (M)	*Citrus aurantifolia* (Christm. and Panzer) Swingle [AA 6603] (fruit)	Salt	Juice	Spoonful	Badigeonnage	3 times/day	At will	5
	Détin (F)	*Elaeis guineensis* Jacq. [AA 6559] (fruit)	Petroleum	Decoction+ extraction	-	Badigeonnage	Twice a day	At will	3
	-	*Pteleopsis suberosa* Engl. and Diels [AA 6571] (bark)	Pierre noire	Maceration	At will	Oral	-	At will	10
	Detin+ tchiayo (F)	*Elaeis guineensis* Jacq. [AA 6559] (fruit)+ *Ocimum gratissimum* L. [AA 6581] (leaves)	Sulphur+salt	Decoction and extraction+ trituration	-	Badigeonnage	Twice a day	At will	6

P=Peulh, F=Fon, I=Idaatcha, M=Mina

**Figure-3:**
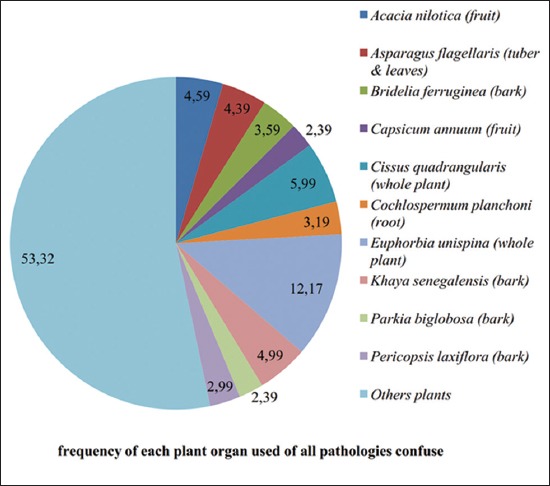
Organ used on each plant to control livestock viral disease.

The most harvested organs on the plants reported in this survey were the barks (36%); the leaves (17%), the whole plants (17%), the fruits (16%), and the roots (6%). Other parts (seeds, stem, tuber, and stubble) represent 8% of the total organs used to prepare the different remedies. Regarding each plant used, whole plant of *E. unispina* were frequently reported by the breeders (Figure-3).

Our survey revealed that maceration of the fruit of *Acacia nilotica* was most used by the breeders, followed by the bark of *Khaya senegalensis* soaked in ox urine to treat cattle foot and mouth disease. To control Newcastle disease in poultry, whole plant of ­*E. ­unispina* were most frequently used, followed by the bark of *Bridelia ferruginea*. The used form of these plants was maceration. African swine fever was treated using soaked root of *Cochlospermum planchonii*. Likewise, the whole plant of *Ageratum conyzoides* was given to animals as fourrage by the breeders to control it. About pseudorinderpest, the maceration of tuber and leaves of *Asparagus flagellaris* was used. The whole plant of *Cissus quadrangularis* soaked and associated with leaves of *Gnidia kraussiana* was also used. Concerning fowl pox, black stone added to the extract obtained from the maceration of bark of *Pteleopsis suberosa* was frequently used by the breeders.

Several plants were indicated for more than one disease in this study. There are namely *Momordica charantia* and *C. quadrangularis*. *M. charantia* is used by farmers to treat Newcastle disease, African swine fever, pseudorinderpest, and fowl pox. *C. quadrangularis* was also used to prevent and treat Newcastle disease, African swine fever, pseudorinderpest, and fowl pox. Both plants were used respectively in the treatment of 4 and 5 animal viral diseases. Similarly, other plants were used to fight two different diseases: It’s the case of *Anogeissus leiocarpa, Cassia sieberiana, K. senegalensis, L. acida, Pericopsis laxiflora, Parkia biglobosa, Sorghum bicolor*, and *Vitellaria paradoxa* to fight foot and mouth disease and Newcastle. ­*A. flagellaris, B. ferruginea, E. ­unispina*, and *G. kraussiana* were used not only to fight Newcastle disease, but they were also used in the preparation of remedies against the pseudorinderpest. Apart from Newcastle, fowl pox was also treated with herbal products such as *Capsicum* *annuum, Ocimum gratissimum, P. suberosa*, and *Vernonia amygdalina*. Regarding of *C. planchonii*, it was used in the preparation of remedies to fight African swine fever and Newcastle disease. Moreover, some plants were used to treat only one viral animal pathology. Among these plants, *Manihot esculenta*, *Pterocarpus erinaceus*, *Saba senegalensis*, and *Terminalia mollis* were used to fight foot and mouth disease. About Newcastle disease, *M. indica*, *Mitracarpus hirtus*, *Moringa oleifera*, *Nicotiana tabacum, Ocimum basilicum; Piper guineense*, and *Sapium grahami* were used in the preparation of remedies. For African swine fever, *Adansonia digitata*; *A. conyzoides; Cussonia arborea*, and *Datura metel* were quoted by breeders for the prevention and treatment of this disease. *Ficus sur* and *Sida garckeana* were mentioned in the preparation of remedies to treat only the pseudorinderpest. Finally, to fight fowl pox, alone, two medicinal plants (*Citrus aurantifolia* and *Elaeis guineensis*) were reported by breeders. Tables-2 to 6 present, respectively, the remedies used for the treatment of these viral diseases. For each of them, the local name and the scientific name of the plant used for treatment, plant organs used, the method of preparation, dosage, route of administration, time of treatment and the numbers of citations were presented.

## Discussion

Viral ailments have high medical and economic importance. During this study, the main viral diseases of livestock that the breeders controlled using medicinal plants were foot and mouth disease, Newcastle disease, pseudorinderpest, African swine fever and fowl pox. According to a study undertaken by Livestock Direction [15], these diseases are serious livestock constraints in a different part of the area study. In Africa, infectious coryza, fowl pox, infectious bursal disease and Newcastle disease are the most widespread infectious diseases in family poultry [2,16]. Also, in many parts of the world, Foot and mouth disease is a highly infectious viral disease of cattle, pigs, sheep, goats, and artiodactyls wildlife species [17]. The use of antiviral synthetic drugs is often unsatisfactory and limited. Mutant viruses’ resistance to the existing antiviral agents arises on treatment [18], and thus contributed to ailments emergences. To remedy this, breeders with the support of the veterinary services make use of the common conventional drugs which are inaccessible to small breeders usually poor. Therefore, medicinal plants have become their favorite [19]. Various studies have shown that different areas in different parts of the world demonstrated the existence of the considerable amount of indigenous ethnomedicinal knowledge [20]. In the ethnobotanical survey conducted in the eight agroecological zones of Benin, 57 medicinal plants were reported to treat or control some common viral diseases in livestock. In Jos, Plateau state, Nigeria, a total of 64 medicinal plant species were reported in the treatment of viral infections [6] among which seventeen species were identified in this study. The medicinal plant species reported in this investigation are also used in other parts of Africa. Among the total of 57 medicinal plant documented in this study, three were mentioned in Wonago, Ethiopia [21]. Some of the plants that were reported were well-known while others were known by a few people. Our study complements existing studies but also extends them for all the eight agroecological zones in the country.

Thus, in this study, 25 medicinal plants were identified for the treatment of foot and mouth disease of which only *K. senegalensis* is reported in Burkina [22]. As a consequence, new plants have been identified in this study. Apart from these plants, some breeders have reported the use of macerated leaves of *Tetradenia riparia* which is spread on the wounds; the use of the powder made from peelings of *Musa paradisiaca* and *Musa sapientum* associated with *N. tabacum* leaves and the oral administration of the bark and roots decoction of *Piliostigma reticulatum*, *K*. *senegalensis*, and *Lonchocarpus laxiflorus* [22,23]. The use of branches of *Azima tetracantha* was reported while *Tinospora cordifolia* smoked resin is used to treat ulcers of the mouth [24]. Similarly, breeders use the sap of the roots of *Sambucus wightiana* [25]. To deal with this disease, three plants (*C. arborea*, *Lagerstroemia microcarpa*, *Syzygium* *cumini var.cumini*) was commonly used in sheepfold [26]. *C. quadrangularis*, *Wrightia tinctoria*, *Vitex* *negundo*, and *Piper nigrum Allium sativum* were identified in the treatment of foot and mouth disease in Rajasthan in India [27]. Similarly, in Ganjam District of Orissa in India, the use of *N. tabacum*, *Semecarpus anacardium*, *Terminalia chebula*, *Acacia catechu*, *M. indica*, *Allium cepa*, *Picrorhiza kurroa*, *Lyonia ovalifolia*, *Juglans regia*, and *Chenopodium ambrosioides* applied externally or as a drink were reported to fight against treatment of foot and mouth disease [28]. The non-use of these plants by the surveyed farmers in the preparation of different remedies may be related to either their ignorance or their absence in the study areas. Similarly, anthropogenic impacts on savannas and forests for the benefit of croplands could be one of the main reasons.

Regarding Newcastle disease recognized as one of the major avian pathology, 32 medicinal plants have been inventoried during this ethno-pharmacological study. Among these plants, *K. senegalensis* and *V*. *amygdalina* were also quoted by farmers in Nigeria [29]. The availability of these plants is certainly linked to their extension and *V. amygdalina* cultivable aspect. This implies the list of 30 new plants in Benin. In Nigeria, Musa *et al*. [29] also report the use of *Solanum nodiflorum*, *Capsicum frutescens*, and *D. metel* by breeders to remedy it. According to Maroyi [30], breeders use *Sesamum angustifolius* fruits to fight against Newcastle disease in Zimbabwe. In Kenya, Okitoi *et al*. [31] also reported the use of the extracts of *Aloe vera*. Poultry farmers frequently use *Capsicum annum* mixed with the ash in water to deal with the disease in Uganda [32].

As for African swine fever, 7 plants had been quoted by breeders who make use of traditional veterinary medicines. Among those plants, *A. digitata*, *A. conyzoide*, *C. quadrangularis*, and *D. metel* are also used to fight against foot and mouth disease and Newcastle [27]. *C. planchonii* is also used to fight against gastro-intestinal infections [11] and Newcastle disease. For this disease that decimates flocks and for which there is no treatment, there is simply no available scientific studies to remedy them.

About pseudorinderpest, which is a major concern for goats and sheep farmers, eight useful medicinal plants have been reported as controlling it. As African swine fever, the traditional treatment techniques of the pseudorinderpest by plants have not been explored so far. However, several extracts of *Combretum paniculatum* tested *in vivo* on some sheep experimentally infected revealed that aqueous and acetone extracts of the leaves of the plant are palliative for goat plague [33].

Finally, with regard to the fowl pox, 8 plants were also identified. Among these plants *C*. *annuum* was also quoted by Lagu and Kayanja [32]. However, *V. amygdalina* have not been mentioned in the most recent work by other authors. According to Buzza and Wamuhehe [34], the use of plants and plant extracts in the control of viral diseases was effective but neglected. Some works are carried out to verify the effectiveness of certain plant species against the fowl pox. Thus, according to Mabiki *et al*. [35], *Synadenium glaucescens* can be used to fight against the disease. Also, Das [28] has reported for the same pathology the use of *Hygrophila auriculata*. The present study showed that bark, leaves, whole plant and roots are indeed the most commonly used medicinal plant parts. This is in line with the finding presented by [6,36,37], respectively, in Nigeria, in Pakistan, and in Ethiopia. However, this is contrary to the finding of [38] in China who reported seeds as the common part used on medicinal plants. In a finding for Ohemu *et al*. [6] in Jos, Plateau state of Nigeria, all the families reported in our study were also mentioned, but the families of Leguminosae-Mimosoideae and Leguminosae-Papilionoideae were not mentioned in their research. These results provide an indication that study area has a rich diversity of ethnoveterinary medicinal plants and indigenous knowledge.

The study also revealed that family Leguminosae-Mimosoideae (10.53%) took the lead in the study areas. Although, recent research in Ethiopia reported that family Asteracea was the highest used to treat ailments [39]. This difference may be due to the fact that their survey was not narrowed to viral diseases but on medicinal plants used in all animal diseases.

Bark was most commonly used for the medicinal purpose than the other plant parts in the study areas. This was followed by leaves, whole plants, fruits, and roots. Many studies conducted in many parts of the world showed that plant leaves are used more than the other parts. For example, in Tirunelveli hills of Western Ghats in India, leaves are sometimes used in combination with other plant parts [40]. The results from this preliminary investigation on viral diseases provide evidence of the importance of ethnopharmacology as a guide to the screening of biologically active plant materials. However, it is possible that these plants may contain some bioactive secondary metabolites that work against viral related infections. Then, it is important to note that it does not necessarily mean that the most mentioned plants are the most effective as only efficacy experiments can determine that.

## Conclusion

The results of this study show that Benin is a major granary of medicinal plants. The breeders in Benin possess rich ethnoveterinary knowledge on medicinal plants and their uses in the treatment of livestock. A total of 57 medicinal plants have been inventoried to fight against five major viral diseases as African swine fever, Pseudorinderpest and Foot and mouth disease. The common plants used to treat viral disease in general were *E. unispina*, *E. poissonii*; *L. acida*; *M*. *indica*. The most harvested organs on the plants reported in this survey were the barks, the leaves and the whole plants. The use of medicinal plants as the therapeutic alternative is well known to the population beside conventional veterinary medicine. Hence, it is necessary to acquire and preserve this traditional system of medicine by proper documentation and identification of specimens. This survey complements the ongoing investigation of different medicinal plants from Benin. It would be important to expand this ethno-pharmacological investigation to other diseases category to better develop our indigenous resources and further scientific evaluations by phytochemical experimentation to determine their effectiveness is needed.

## Authors’ Contributions

Kpodékon Marc is my supervisor and assisted in building the conceptual framework, the development and reviewing of this manuscript; Ogni Clément: This work is part of my PhD thesis; Dassou Hospice help in data collection and in plants identification; Dougnon Jacques, Boko Cyrille, Koutinhouin Bénoît, Goussanou Judicaël and Akoegninou Akpovi conducted the fieldwork; Youssao Issaka performed statistical analyses of the data. All authors participated in the writing and revision process read, discussed and approved the final manuscript.
